# Scrub Typhus and Bilateral Lateral Rectus Palsy: An Uncommon Manifestation

**DOI:** 10.7759/cureus.69891

**Published:** 2024-09-21

**Authors:** Sonali Ghosh, Ranabir Ghosh, Sourav Das Choudhury, Kaushik Ghosh

**Affiliations:** 1 Department of Emergency Medicine and Critical Care, Institute of Post-Graduate Medical Education and Research (IPGMER) and Seth Sukhlal Karnani Memorial (SSKM) Hospital, Kolkata, IND; 2 Anaesthesiology, Murshidabad Medical College and Hospital, Berhampore, IND; 3 Medicine, Nibedita Health Care, Berhampore, IND; 4 Medicine, Murshidabad Medical College and Hospital, Berhampore, IND

**Keywords:** bilateral abducens nerve palsy, diplopia, neurological manifestations, orientia tsutsugamushi, scrub typhus

## Abstract

Scrub typhus is a zoonotic disease that typically presents as an acute febrile illness. It is caused by the rickettsial organism *Orientia tsutsugamushi*, which is prevalent in the Asian region. The clinical manifestations of scrub typhus can vary significantly, ranging from mild febrile illness to severe multiorgan dysfunction syndrome. While the clinical presentation can be diverse, abducens nerve palsy is an extremely uncommon complication reported. This report discusses a 14-year-old previously healthy girl, who exhibited sudden onset of bilateral abducens nerve palsy, accompanied by headache and fever, but without the characteristic skin lesion known as "eschar." After ruling out common infectious diseases and other potential causes, she was ultimately diagnosed with scrub typhus complicated by abducens nerve palsy, which showed full recovery with doxycycline treatment.

## Introduction

Scrub typhus is a rickettsial infection caused by Gram-negative *Orientia tsutsugamushi* transmitted by infected larval mites, known as chiggers, which leave a characteristic eschar at the bite site sometimes. In endemic regions, outbreaks can occur during the rainy season as chiggers are more active [1]. Although scrub typhus most commonly presents as acute febrile illness with fever, headache, myalgia, and rashes, it is often overlooked because of nonspecific clinical presentation, lack of diagnostic facilities, and low index of suspicion by clinicians. Symptoms may include coughing, lymphadenopathy, and gastrointestinal symptoms. In case of delayed treatment, scrub typhus can lead to severe complications, such as pneumonia, acute respiratory distress syndrome, acute kidney injury, hepatitis, splenic infarct, gastrointestinal bleeding, myocarditis with shock, and neuropsychiatric manifestations including multi-organ failure [2]. Central nervous system involvement may cause meningoencephalitis, confusion, seizures, cranial nerve palsies, intracranial haemorrhage, cerebellitis, Guillain-Barre syndrome, acute transverse myelitis, neuroleptic malignant syndrome, and coma. However, clinical involvement of bilateral abducens nerve is rarely reported [3]. We report a case of scrub typhus infection manifesting with bilateral lateral rectus palsy in a tertiary care hospital in Eastern India that was successfully cured.

## Case presentation

A 14-year-old girl from Murshidabad, West Bengal, presented with high-grade, continuous fever lasting for 15 days, accompanied by severe headaches that began around the same time. Besides the fever and headache, the patient eventually developed double vision and decreased distance vision in both eyes.

According to the patient, the fever subsided with medication, but the headache persisted. The patient denied any history of altered sensorium, convulsions, or vomiting. Additionally, the patient had no history of tuberculosis.

**Figure 1:**
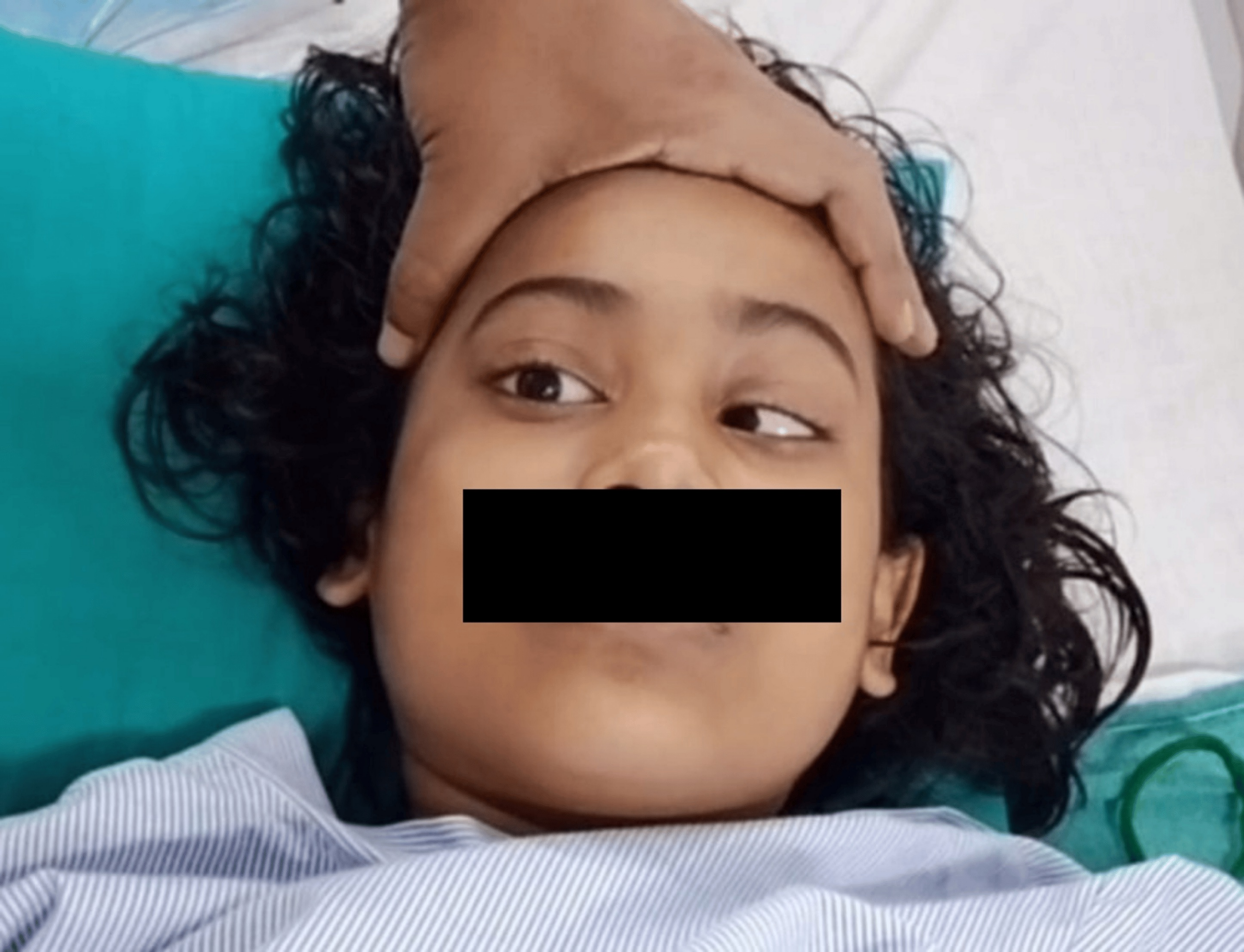
Right-sided lateral rectus palsy

**Figure 2:**
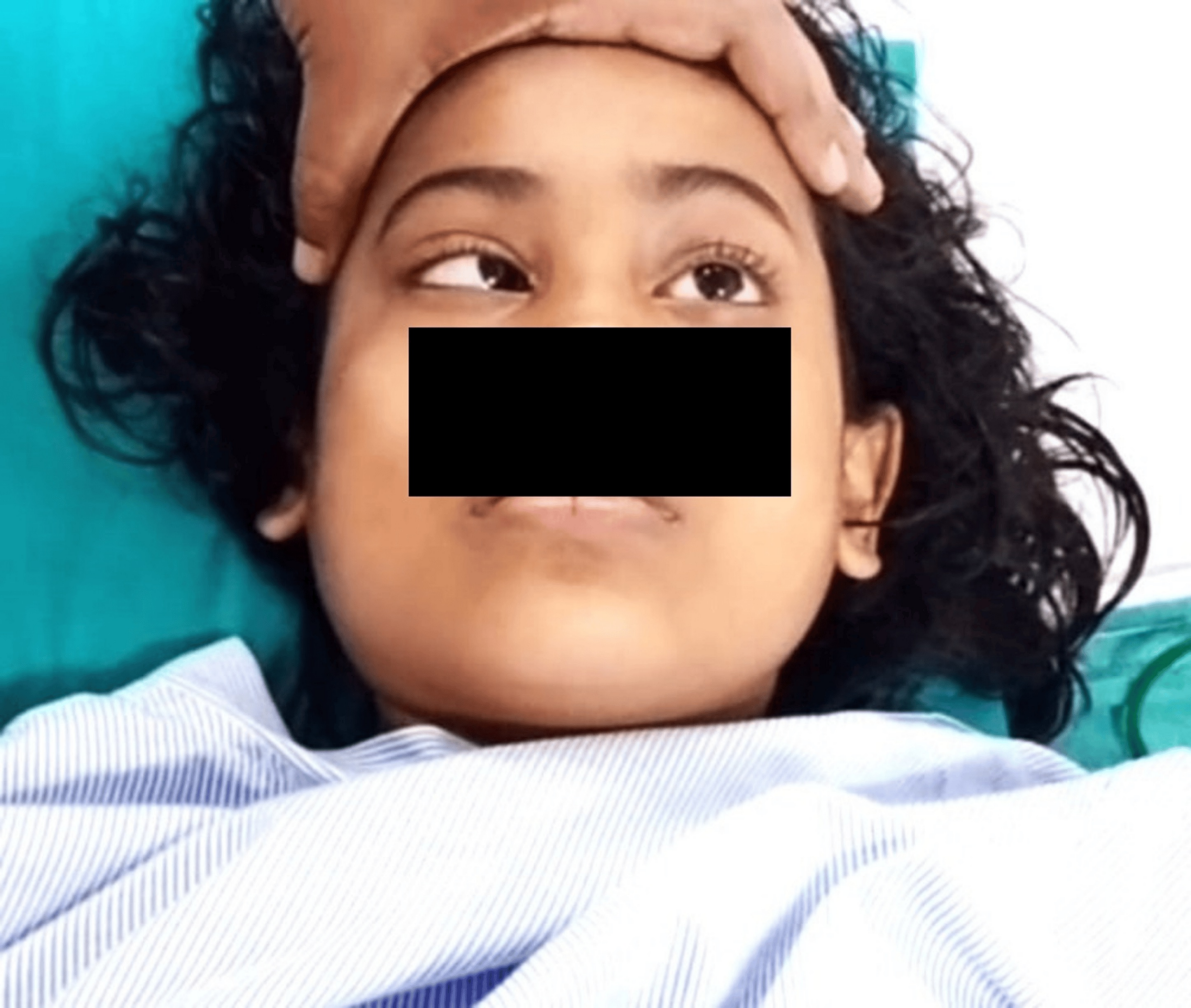
Left-sided lateral rectus palsy

Upon examination, the patient was febrile with a normal sensorium. The pulse rate was 98 beats/min, blood pressure was 100/70 mmHg, oxygen saturation was 98% in room air, and respiratory rate was 20 breaths/min. The respiratory, cardiovascular, and abdominal examinations revealed no abnormalities. On neurological examination, the patient’s higher function was normal. No neck rigidity or positive meningeal signs were observed. Cranial nerve examination revealed bilateral lateral rectus palsy (Figures 1, 2). Her bedside visual acuity and colour vision were normal. Ophthalmoscopic evaluation revealed normal fundi.

Lab investigations (Table 1) showed the haemoglobin level was 10.2 gm%, total leukocyte count was 8800/cumm, platelet count was 2.85 lacs/cumm, packed cell volume was 30.8%, and erythrocyte sedimentation rate was 24 mm/h. The peripheral blood smear revealed normocytic normochromic anaemia. Liver function test revealed a serum total bilirubin level of 0.6 mg/dL; albumin level of 3.4 gm/dL; globulin level of 3.2 gm/dL; serum glutamic-oxaloacetic transaminase and serum glutamic-pyruvic transaminase levels of 54 U/L and 64 U/L, respectively; and blood sodium, potassium, chloride, urea, creatinine, and C-reactive protein levels of 136.9 mmol/L, 4.16 mmol/L, 99.2 mmol/L, 14.2 mg/dl, 0.6 mg/dl, and 55.78 mg/dL, respectively. Blood serological tests for hepatitis B surface antigen, anti-hepatitis C virus, and human immunodeficiency virus 1 and 2 were negative. Malarial parasite slides and dual antigen test results were negative. Enzyme-linked immunosorbent assays (ELISAs) for dengue non-structural protein-1 antigen and anti-dengue immunoglobulin M (IgM) and immunoglobulin G were also negative. The Widal test and typhi dot IgM antibody test were negative. ELISA for scrub typhus IgM antibody was positive. Chest radiography, abdominal ultrasonography, and non-contrast brain computed tomography showed no abnormal findings. Gram staining and acid-fast bacteria (AFB) staining of the CSF were negative. CSF culture showed no growth after 48 h of aerobic incubation at 37°C. The CSF was negative for Mycobacterium tuberculosis DNA load. The CSF findings were suggestive of meningitis (possibilities in order of preference pyogenic meningitis on treatment and viral meningitis).

**Table 1:** Laboratory results CSF- Cerebrospinal Fluid

Test	Result	Unit	Reference value
Hemoglobin	10.2	gm% (gm/dL)	11.5-15.2
Total leucocyte count	8800	/cumm	3500-10000
Platelet count	2.85	lacs/cumm	1.5-4
Packed cell volume	30.8	%	35-46
Erythrocyte sedimentation rate	24	mm in 1^st^ hour	0-20
Serum total bilirubin	0.6	mg/dL	Upto 2
Serum albumin	3.4	gm/dL	3.5-5.2
Serum globulin	3.2	gm/dL	1.2-3.3
Serum glutamic-oxaloacetic transaminase	54	U/L	Upto 40
Serum glutamic-pyruvic transaminase	64	U/L	Upto 41
Serum sodium	136.9	mmol/L	135-155
Serum potassium	4.16	mmol/L	3.5-5.5
Serum chloride	99.2	mmol/L	98-107
Serum urea	14.2	mg/dL	15-40
Serum creatinine	0.6	mg/dL	0.6-1.2
C-reactive protein	55.78	mg/dL	0-6
CSF total cell count	100	/cumm	<5
CSF sugar	68	mg/dL	50-80
CSF protein	64.7	mg/dL	23-38
CSF chloride	110	mmol/L	115-130
CSF adenosine deaminase	6.7	U/L	0-2.5

**Figure 3:**
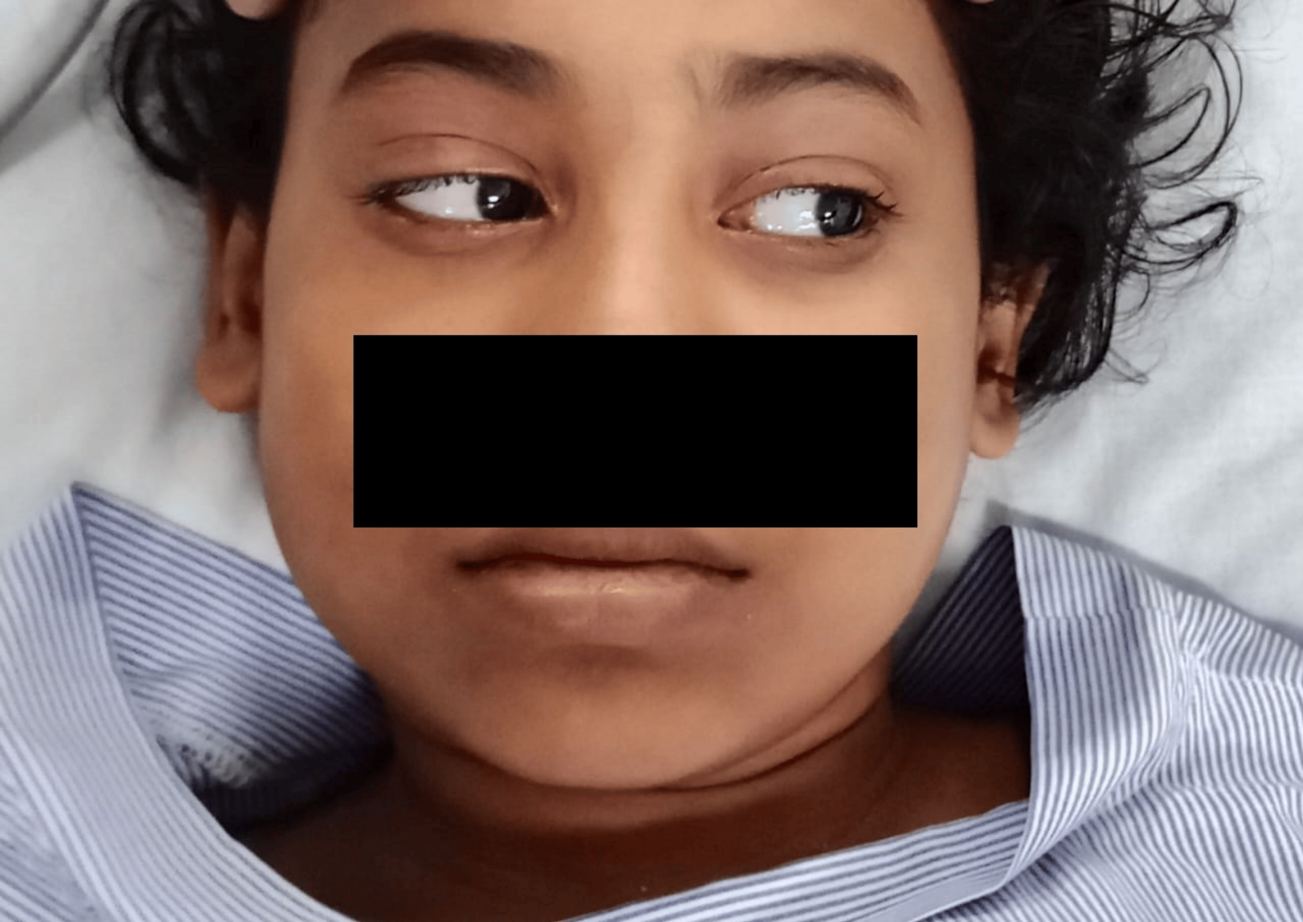
Fully recovered ocular movements

The patient was treated with a full course of parenteral doxycycline, other supportive medications, and ocular movement exercises. The fever subsided two days after admission, and the bilateral lateral rectus palsy fully recovered in the following five days (Figure 3). She was discharged after seven days, with advice to follow up in the outpatient department after two weeks.

## Discussion

Scrub typhus is a bacterial infection caused by* Orientia tsutsugamushi* transmitted to humans through the bites of infected chiggers. Antigenically, this Gram-negative coccobacillus differs from the typhus-causing Rickettsia family of bacteria [1,4]. It primarily targets endothelial cells lining the blood vessels, leading to systemic vasculitis and inflammation. *Orientia tsutsugamushi* escapes host defence with the help of phospholipase A2 and invades the host microcirculation, resulting in a wide range of clinical manifestations, including central nervous system (CNS) involvement. Meningoencephalitis is the most common neurological manifestation [4].

An atypical presentation of scrub typhus is bilateral lateral rectus palsy [3]. The lateral rectus muscle is responsible for the outward movement of the eye. Thus, paralysis or weakness of the lateral rectus can result in double vision, particularly if looking towards the affected side.

Although there is no direct link between scrub typhus and lateral rectus palsy, scrub typhus can result in neurological complications, including bilateral or unilateral abducens nerve palsy. The bacteria invade endothelial cells, resulting in inflammation and damage to the blood vessels, thus allowing bacteria and inflammatory mediators to enter the CNS and disrupt the blood-brain barrier. Excessive production of cytokines (interleukin 6 (IL-6), tumor necrosis factor α (TNFα)) in response to infection can lead to systemic inflammation and CNS manifestations. Vasculitis and inflammation can cause neuronal damage due to reduced blood flow to the brain [4,5].

This case demonstrates an atypical clinical presentation of scrub typhus in endemic areas. Notwithstanding shared signs and symptoms of other “feverish” conditions, scrub typhus has become a diagnostic problem for doctors [5]. This case presented with an atypical neurological manifestation of bilateral lateral rectus palsy, suggesting a broad spectrum of clinical symptoms of scrub typhus. Fever is accompanied by headache, myalgia, and ocular muscle paralysis, which are rare symptoms that should not be overlooked [6]. This kind of neurophysiological presentation can progress to severe disability if immediate diagnosis and proper treatment are not provided.

The presence of scrub typhus IgM antibodies in the patient’s serum was a decisive factor in the diagnosis of *Orientia tsutsugamushi* infection. Thus, serological testing is compulsory for diagnosis, regardless of the presence of eschar [7]. This specific dermatological lesion is present only in limited instances but is not obligatory for all cases of eczema [8]. In our case, the CSF test showed lymphocytic dominance, an increased level of protein, and the expected level of glucose that could be observed in the materials. The culture tests and Gram-staining were negative for the presence of bacteria, confirming the non-bacterial nature of typhus. Therefore, meningitis may be associated with fever. Moreover, laboratory tests corroborated the clinical diagnosis of scrub typhus, hence highlighting the value of a comprehensive assessment, even under atypical circumstances.

Early diagnosis of scrub typhus, even in unusual cases, is of vital importance in the timely initiation of antibiotic therapy, such as doxycycline [9]. Late diagnosis or failure to diagnose may cause severe conditions, such as multi-organ failure and death. Healthcare professionals should always be alert to the possibility of scrub typhus, which should be considered in the differential diagnosis of fever-causing diseases [10]. Healthcare professionals should be vigilant, particularly in endemic areas. In the early phase of infection, normochromic-normocytic anaemia may be present, accompanied by leukocytosis, suggestive of systemic infection. This haematological feature develops as a result of the host immune response to pathogens. Furthermore, an increase in liver enzyme levels indicates a possible liver effect. The liver is among the organs most frequently affected psychologically because of the systemic nature of this infection, leading to multi-organ system involvement [11]. Liver function derangements, caused by transaminases, can help in the diagnosis of the infection and following its course in patients.

Prompt initiation of appropriate antibiotic therapy along with supportive care is crucial, and favourable outcomes can be achieved. Prompt antibiotic therapy is essential to highlight the essence of fast diagnosis and increased levels of clinical efficiency in areas with inadequate special diagnostic tools owing to resource constraints [12]. This case highlights the need for healthcare workers to increase their awareness of different forms of scrub typhus.

Diagnosing scrub typhus with this atypical presentation is challenging. Therefore, clinicians should consider the possibility of scrub typhus infection in these endemic regions, along with other probable differentials.

Treatment usually involves supportive care, ocular movement exercises, and monitoring for complications.

## Conclusions

Scrub typhus displays the participation of various organs and can cause difficult diagnosis. The clinical presentation of scrub typhus can vary from mild symptoms to severe complications, depending on the system involved and early diagnosis.

Practitioners working in endemic settings with high disease loads should be more cautious and alert about all diagnostic and definitive observations. In several cases, bilateral lateral rectus palsy is atypical; therefore, an index of high suspicion is required for prompt recognition and correct treatment.
